# A Case of 5-Fluorouracil-Induced Coronary Artery Vasospasm in a Patient With Salivary Gland Cancer

**DOI:** 10.7759/cureus.10887

**Published:** 2020-10-10

**Authors:** Iva N Dimitrova

**Affiliations:** 1 Cardiology, St. Ekaterina University Hospital, Medical University of Sofia, Sofia, BGR

**Keywords:** 5-fluorouracil, myocardial infarction, coronary vasospasm, chemotherapy, salivary gland cancer

## Abstract

5-Fluorouracil (5-FU), a pyrimidine analogue, is widely used in different chemotherapy regimens with established indications for the treatment of gastrointestinal, breast, head, and neck tumors. Various prospective studies including randomized controlled trials and retrospective reviews have shown a wide range of reported incidence of cardiotoxicity related to 5-FU use. This incidence is dependent on drug regimen, doses, concomitant therapy, patients’ clinical characteristics, and risk factors. Herein, we present a clinical case of coronary vasospasm mimicking ST-elevation myocardial infarction during a 5-FU infusion for salivary gland cancer. Cardiologists and oncologists must keep in mind the potential life-threatening side effects of 5-FU on the heart and they must be familiar with the risk factors for their occurrence and their management strategies.

## Introduction

Cancer is the second leading cause of death worldwide. The World Health Organization predicts that there will be a rise in cancer incidence by 70% over the next 20 years [1]. 5-Fluorouracil (5-FU) is a chemotherapeutic drug used in the treatment of gastrointestinal, esophageal, renal, bladder, breast, head, and neck tumors. It can cause cardiotoxic effects including chest pain, myocardial infarction, arrhythmias, myocarditis, pericarditis, heart failure, and death [2]. The reported incidence is variable, between 0-20%; the most frequent symptom is chest pain (0-18.6%), reported mortality varies from 0 to 8% [3].

Possible factors increasing 5-FU cardiotoxicity are: underlying cardiovascular diseases, traditional risk factors like hypertension, hyperlipidemia, smoking; continuous vs bolus regimen of application; concomitant or previous radiation therapy; and simultaneous administration of another cardiotoxic drug [2,4]. Further investigations on risk stratification are needed because of the lack of definitive data and the controversial results of previous studies.

Suggested mechanisms of 5-FU toxicity are coronary vasospasm (endothelial or smooth muscle dysfunction), autoimmune-mediated injury of the myocardium, thrombogenic effects or thrombus formation, direct myocardial toxicity causing necrosis, accumulation of metabolites, altered red blood cell morphology, and impaired oxygen-carrying capacity [2,5].

In this report we present a case of suggested coronary vasospasm, mimicking acute myocardial infarction in a 67-year-old man, during 5-FU infusion for salivary gland cancer.

## Case presentation

**Figure 1:**
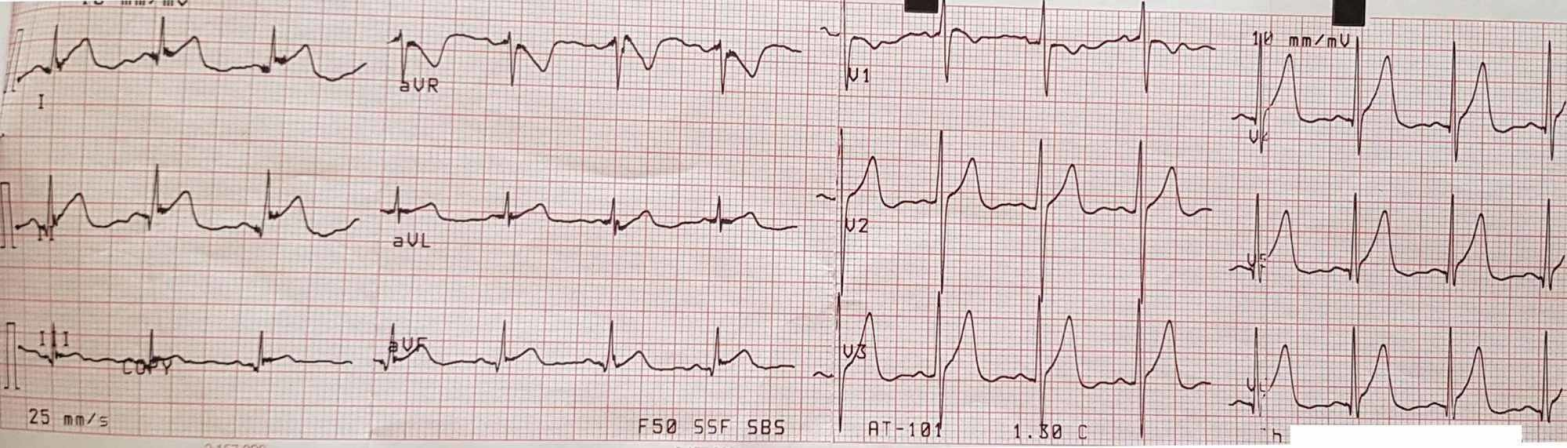
ST elevation on leads – I, aVL, II, III, aVF, hyperacute T waves V2-V6 with reciprocal changes in aVR during an anginal attack

A 67-year-old man was transferred from the Oncology Department in our Cardiology Intensive Care Unit (ICU) with suspected ST-elevation myocardial infarction. He had a history of mucoepidermoid carcinoma of the left salivary gland (T2N1M0), excised with lymph node dissection and treated with radiotherapy eight months earlier. Because of cancer recurrence, a chemotherapy regimen was administered - 5-FU continuous intravenous infusion, cetuximab and cisplatin. On the third 5-FU application, during an infusion the patient suffered from continuous chest pain. The electrocardiogram (ECG) showed ST elevation on leads I, aVL, II, III, aVF, hyperacute T waves V2-V6 with reciprocal changes in aVR (Figure 1).

**Figure 2:**
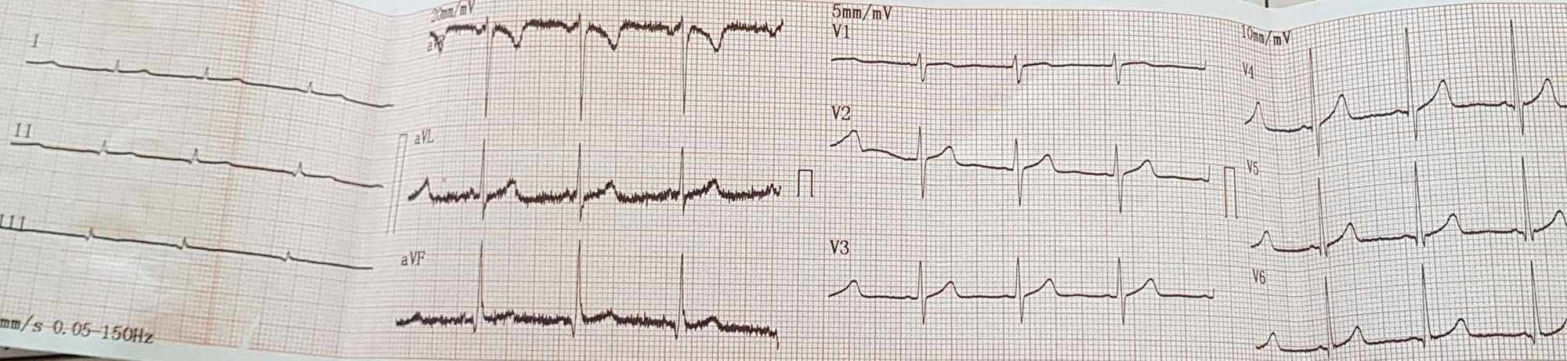
Complete resolution of the ST changes after sublingual nitroglycerin administration.

The 5-FU infusion was discontinued, and sublingual nitroglycerin administered, with pain relief occurring after 10 minutes. In cardiology ICU ECG showed complete resolution of the ST changes observed earlier and there was complete pain relief (Figure 2).

**Figure 3:**
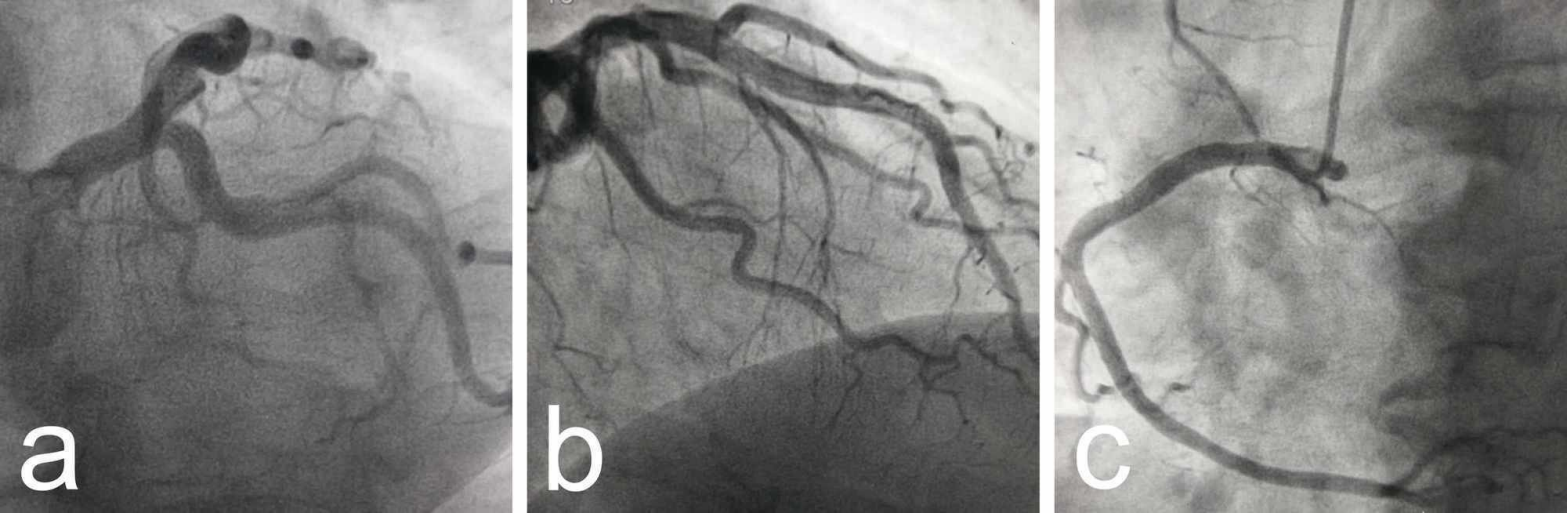
a, b) Normal appearance of left coronary system during angiography; c) Normal appearance of right coronary artery.

The patient had no history of heart disease. He had arterial hypertension, dyslipidemia, and smoking as risk factors. Samples for myocardial necrosis markers - creatine phosphokinase, creatine phosphokinase-MB and troponin T were within normal ranges. Although the echocardiography showed no wall motion abnormalities, preserved left ventricular ejection fraction, and mild mitral regurgitation, coronary angiography was performed which revealed non-obstructive coronary artery disease (Figure 3 a-c).

During hospital stay, the patient had no complaints, his ECG, cardiac enzyme levels, and echocardiography remained normal. Suggesting the relationship between 5-FU administration, typical chest pain, and reversible ECG changes probably due to coronary vasospasm in the context of chemotherapy cardiotoxicity, we advised the oncologists to consider therapeutic regimen modification, if not possible - strict ECG monitoring during the 5-FU infusion and prophylactic use of calcium channel blocker (CCB) and nitrate. The patient was discharged on the fourth day after the procedure.

## Discussion

Cardiovascular diseases and cancer are a leading cause of morbidity and mortality worldwide. Cardiotoxicity is an important and sometimes life-threatening effect of some chemotherapeutic drugs. 5-FU is in wide use for the treatment of solid tumors and is the second most cardiotoxic chemotherapeutic drug [6]. The reported wide range of its incidence, the controversial risk profile reported in studies, the numerous proposed mechanisms of 5-FU-induced cardiotoxicity, and the lack of established guidelines for treatment define the need for further research.

Coronary vasospasm is the most established theory for 5-FU-related cardiotoxicity. Despite the evidence of significant ST changes and, sometimes, elevated troponin levels, often no coronary artery disease is observed in coronary angiography or computed tomography [7-9]. Coronary artery and brachial artery vasospasm following 5-FU infusion has been observed in angiography or ultrasound [10-12]. Nitrates and CCB are effective in pain relief and seem to correct ECG changes [5]. This data taken together supports the vasospastic theory, although other pathophysiological mechanisms of 5-FU-induced cardiotoxicity are possible.

In the presented case there were diffuse ECG changes during the pain period that may be a sign of pervasive coronary artery vasospasm rather than vasospasm of an isolated epicardial artery. The discontinuation of 5-FU infusion and the application of sublingual nitroglycerine fully relieved the chest pain and reversed the ECG and echocardiography changes. Management approaches have been suggested for 5-FU induced cardiotoxicity. It is recommended to discontinue 5-FU application or if not possible, rechallenge carefully the treatment with administration of CCB, long-acting nitrates, and close cardiac monitoring [2]. Experimental treatments exploring the protective effect of coenzyme complex or glucagon-like peptide-1 analogues may reveal new options in the management of 5-FU-induced cardiotoxicity [4].

## Conclusions

Clinical manifestations of 5-FU-induced cardiotoxicity are often treatable and reversible but could be life-threatening. This requires patient risk stratification, knowledge of the potential mechanisms, and management strategies. Close collaboration between cardiologists and oncologists is advisable in order to achieve improved clinical outcomes and to reduce potential complications.
